# The Effect of Pegbovigrastim Injection on Phagocytic and Oxidative Burst Activities of Peripheral Blood Granulocytes and Monocytes in Calves Challenged with *Mycoplasma bovis*

**DOI:** 10.3390/pathogens11111317

**Published:** 2022-11-09

**Authors:** Katarzyna Dudek, Ewelina Szacawa, Magdalena Wasiak, Dariusz Bednarek, Michał Reichert

**Affiliations:** 1 Department of Cattle and Sheep Diseases, National Veterinary Research Institute, 57 Partyzantów Avenue, 24-100 Pulawy, Poland; 2 Department of Pathology, National Veterinary Research Institute, 57 Partyzantów Avenue, 24-100 Pulawy, Poland

**Keywords:** *Mycoplasma bovis*, calves, pegbovigrastim, phagocytic activity, oxidative burst, granulocytes, monocytes

## Abstract

*Mycoplasma bovis* (*M. bovis*) is an important pathogen affecting cattle, causing various diseases including pneumonia which mainly occurring in calves. Control of *M. bovis* infections is difficult due to the lack of commercial vaccines in most parts of the world and increasing trends of antimicrobial resistance in field isolates of the pathogen; therefore, it seems reasonable to look for new solutions for the prevention of the infection. Pegbovigrastim is a pegylated form of naturally occurring circulating cytokine in cattle that affects bovine leukocytes and some cell functions. Most studies on pegbovigrastim have focused on reducing the occurrence of mastitis and other diseases occurring during the periparturient period in cows, while this study attempts to use pegbovigrastim in the prevention of respiratory diseases in calves, which are largely caused by *M. bovis*. Based on previous observations on the immunostimulatory properties of pegbovigrastim in cattle, for the first time, the effect of its injection on the number and phagocytic and oxidative burst activities of peripheral blood granulocytes and monocytes in calves experimentally infected with *M. bovis* was investigated. Pegbovigrastim administration in the calves significantly stimulated an increase in peripheral blood granulocyte and monocyte counts and phagocytic activity of the cells, especially granulocytes, which was also generally expressed in the course of *M. bovis* infection. In response to pegbovigrastim administration, a general increase in the oxygen burst activity of the cells was observed. This effect was also shown despite ongoing infection with *M. bovis* which, taken together, may indicate a beneficial effect of pegbovigrastim injection on the immunity of the affected animals.

## 1. Introduction

*Mycoplasma bovis* (*M. bovis*) is a causative agent of many diseases in cattle including pneumonia, arthritis, mastitis, otitis, keratoconjunctivitis, endocarditis, and brain disorders. However, pneumonia caused by *M. bovis* alone or in co-infection with other etiological agents of bovine respiratory disease complex (BRD) is currently a major health problem in calves [1]. Infections with *M. bovis* are diagnosed all over the world where cattle breeding exists, affects all age groups and sectors of cattle, and causes significant economic losses in the cattle industry [2,3]. *M. bovis* is able to modulate the immune response in cattle, including some functions of bovine leukocytes [4,5,6,7]. Control of *M. bovis* infections is difficult due to the lack of commercial vaccines, except in the United States [8,9]. Therefore, the prevention of infections with *M. bovis* still relies on the use of autogenous vaccines produced on a small scale, in combination with maintaining generally understood principles of biosecurity [1,10]. Additionally, due to the increasing resistance of field *M. bovis* isolates to commonly used antimicrobials, it seems reasonable to look for new solutions for the prevention and eradication of the infection [11].

Granulocyte colony-stimulating factor (G-CSF) is a circulating hematopoietic cytokine that plays an important role in granulopoiesis, production, proliferation, and differentiation of neutrophils [12]. Its concentration increases during infection, inflammation, and stress [13]. At an early stage of acute inflammation, G-CSF can regulate the mobilization of neutrophils from bone marrow to blood circulation [14,15]. Conversely, the opposite relationship takes place in the late phase of this inflammation, during which reserves of these cells are created in the bone marrow due to the suppression of rapid neutrophil mobilization mediated by G-CSF [14,16,17,18]. The pegylated recombinant form of bovine G-CSF (PEG bG-CSF), pegbovigrastim, can stimulate the circulating total white cell, neutrophil, and monocyte counts in dairy cows during the transition period [19]. Later data has shown some beneficial effects of PEG bG-CSF administration in grazing dairy cows, reducing the occurrence of clinical mastitis as well as reducing the incidence of subsequent endometritis in cows previously diagnosed with metritis [20]. Some preventive properties of pegbovigrastim administration in dairy cows around the time of calving were also demonstrated in relation to the incidence of post-calving antimicrobial treatments during the first 30 days of lactation [21]. However, studies on calves using pegbovigrastim are very sparse. The stimulating effect of pegbovigrastim on the number of circulating leukocytes in calves was previously demonstrated; however, there is still no information about the alternations in leukocyte function post pegbovigrastim administration under both physiological and pathological conditions [22]. Based on previous observations on the immunostimulatory properties of pegbovigrastim in cattle, the effect of its injection on the number as well as phagocytic and oxidative burst activities of peripheral blood granulocytes and monocytes in calves experimentally infected with *M. bovis* was investigated for the first time.

## 2. Results

### 2.1. Hematology

**Figure 1:**
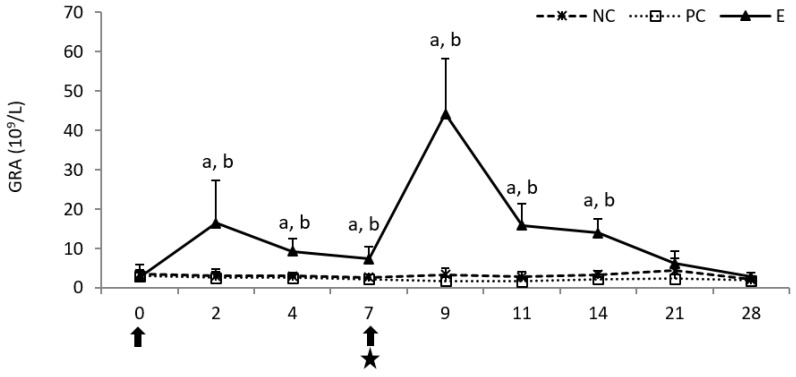
The granulocyte (GRA) count in the peripheral blood of calves after injection of pegbovigrastim and post-challenge with *M. bovis*. E—experimental group; PC—positive control group; NC—negative control group; arrow—pegbovigrastim injection; star—infecting dose of *M. bovis*; a—*p* < 0.05 between the E and NC groups; b—*p* < 0.05 between the E and PC groups.

In the experimental (E) group, a statistically significant increase (*p* < 0.05) in the granulocyte (GRA) count was observed following the first pegbovigrastim injection compared to both controls, which lasted until day 7 of the study. It was intensified following the second injection (on day 9 of the study) and remained statistically significantly higher (*p* < 0.05) than in both controls until day 14 of the study, despite ongoing *M. bovis* infection. Post-infection, in the positive control (PC) group the GRA count was visibly lower than in the negative control (NC) group up to day 21 of the study (Figure 1).

**Figure 2:**
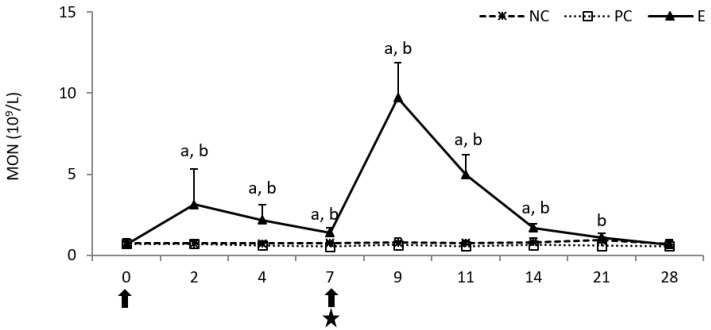
The monocyte (MON) count in the peripheral blood of calves after injection of pegbovigrastim and post-challenge with *M. bovis*. E—experimental group; PC—positive control group; NC—negative control group; arrow—pegbovigrastim injection; star—infecting dose of *M. bovis*; a—*p* < 0.05 between the E and NC groups; b—*p* < 0.05 between the E and PC groups.

Following the first injection of pegbovigrastim, a statistically significant increase (*p* < 0.05) in the monocyte (MON) count was observed until day 7 when compared to both control groups. This increase was intensified following the second injection of pegbovigrastim, despite ongoing *M. bovis* infection, and was statistically significant (*p* < 0.05) when compared to both controls up to day 14 of the study as well as on day 21 of the study when compared to the PC group. Post-infection in the PC group, the MON count was lower than in the NC group until the end of the study on day 28 (Figure 2).

### 2.2. Flow Cytometry

#### 2.2.1. Phagocytic Activity

**Figure 3:**
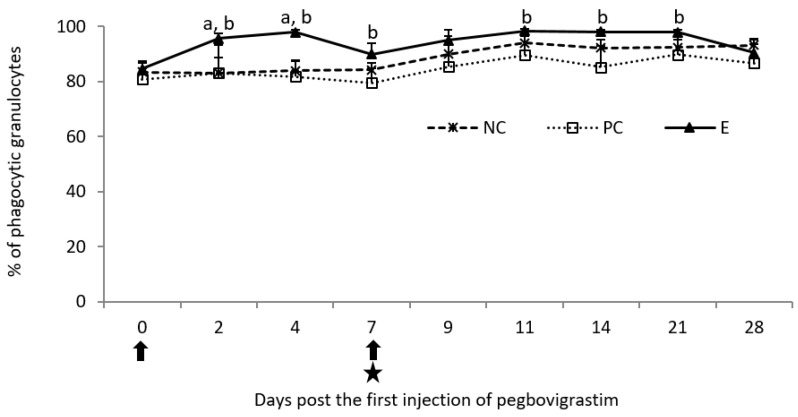
Phagocytic activity of granulocytes expressed as a mean percentage of phagocytic cells in the peripheral blood of calves after injection of pegbovigrastim and post-challenge with *M. bovis*. E—experimental group; PC—positive control group; NC—negative control group; arrow—pegbovigrastim injection; star—infecting dose of *M. bovis*; a—*p* < 0.05 between the E and NC groups; b—*p* < 0.05 between the E and PC groups.

Following the first injection of pegbovigrastim, a statistically significant increase (*p* < 0.05) in the percentage of phagocytic granulocytes was observed when compared to both controls up to day 4 of the study, and additionally on day 7 of the study when compared to the PC group (Figure 3). The increase was still present following the second injection of pegbovigrastim, despite ongoing *M. bovis* infection; however it was statistically significant (*p* < 0.05) on days 11, 14, and 21 of the study compared to the PC group only. Following the infection in the PC group, lower values of this parameter were observed than in remaining groups of calves until the end of the study on day 28 (Figure 3).

**Figure 4:**
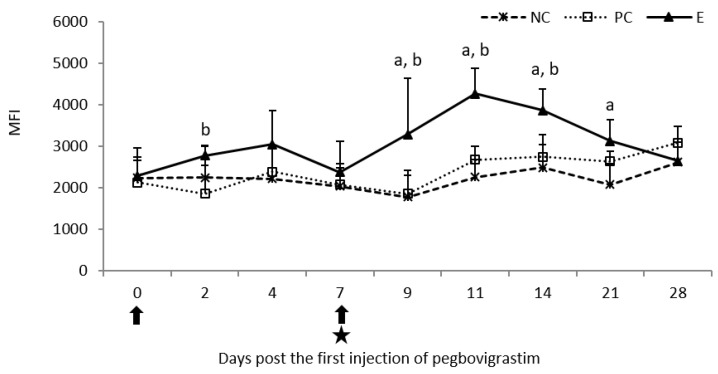
Phagocytic activity of granulocytes expressed as mean fluorescence intensity (MFI) in the peripheral blood of calves after injection of pegbovigrastim and post-challenge with *M. bovis*. E—experimental group; PC—positive control group; NC—negative control group; arrow—pegbovigrastim injection; star—infecting dose of *M. bovis*; a—*p* < 0.05 between the E and NC groups; b—*p* < 0.05 between the E and PC groups.

An increase in the phagocytic activity of granulocytes, expressed as mean fluorescence intensity (MFI), was observed following the first injection of pegbovigrastim and it was statistically significantly higher (*p* < 0.05) than the PC group on day 2. This increase was intensified following the second injection of pegbovigrastim, despite ongoing *M. bovis* infection; it was statistically significant (*p* < 0.05) in the E group on days 9, 11, and 14 of the study when compared to both controls, and additionally on day 21 of the study when compared to the NC group. On day 4 following infection (day 11 of the study), a slight increase in the MFI was observed in the PC group when compared to the NC group which persisted until the end of the study on day 28 (Figure 4).

**Figure 5:**
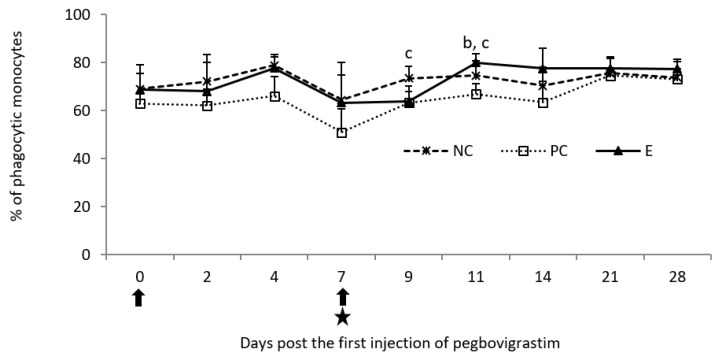
Phagocytic activity of monocytes expressed as a mean percentage of phagocytic cells in the peripheral blood of calves after injection of pegbovigrastim and post-challenge with *M. bovis*. E—experimental group; PC—positive control group; NC—negative control group; arrow—pegbovigrastim injection; star—infecting dose of *M. bovis*; b—*p* < 0.05 between the E and PC groups; c—*p* < 0.05 between the PC and NC groups.

Following the first injection of pegbovigrastim, the percentage of phagocytic monocytes was comparable to the NC group and slightly higher than the PC group until day 7. A transient decrease in this parameter was observed on day 9 of the study following the second injection of pegbovigrastim and infection with *M. bovis*. On that day, the values in the E group were comparable to those in the PC group. However, at that time, statistically significantly lower percentage of phagocytic monocytes (*p* < 0.05) was observed in the PC group when compared to the NC group, and this continued on day 11 of the study. On that day, this parameter was also statistically significantly lower (*p* < 0.05) compared to the E group. On day 14 of the study, a further increase in the percentage of phagocytic monocytes was observed in the E group when compared to both controls and, after this time, the values of this parameter were comparable in all examined groups (Figure 5).

**Figure 6:**
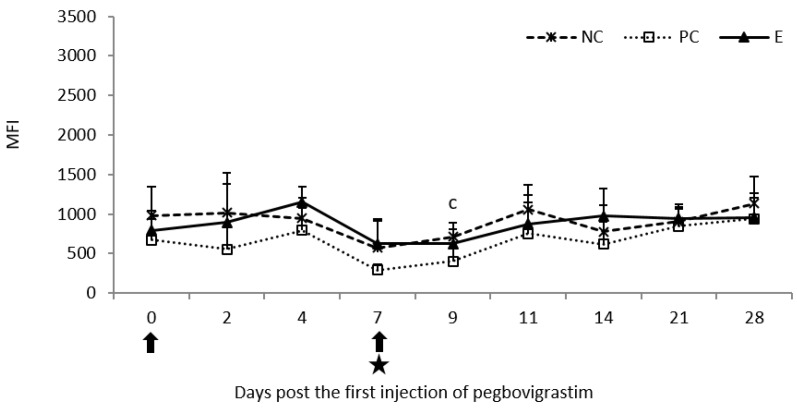
Phagocytic activity of monocytes expressed as mean fluorescence intensity (MFI) in the peripheral blood of calves after injection of pegbovigrastim and post-challenge with *M. bovis*. E—experimental group; PC—positive control group; NC—negative control group; arrow—pegbovigrastim injection; star—infecting dose of *M. bovis*; c—*p* < 0.05 between the PC and NC groups.

Following the first injection of pegbovigrastim, there were no statistically significant differences (*p* < 0.05) in the phagocytic activity of peripheral blood monocytes expressed as MFI when compared to both control groups until day 7. For comparison, on day 2 following the infection in the PC group (day 9 of the study) statistically significantly lower values (*p* < 0.05) of this parameter were observed when compared to the NC group. Apart from this time point, no statistically significant differences (*p* < 0.05) between all examined groups in the MFI were observed until the end of the study, i.e., on day 28 (Figure 6).

#### 2.2.2. Oxidative Burst Activity

**Figure 7:**
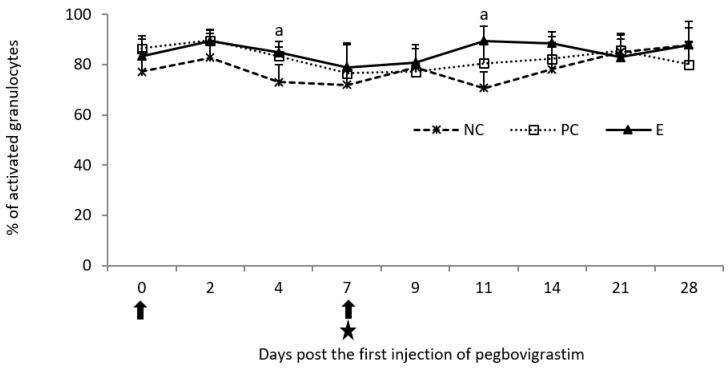
Oxidative burst activity of granulocytes expressed as a mean percentage of cells in the peripheral blood of calves after injection of pegbovigrastim and post-challenge with *M. bovis*. E—experimental group; PC—positive control group; NC—negative control group; arrow—pegbovigrastim injection; star—infecting dose of *M. bovis*; a—*p* < 0.05 between the E and NC groups.

Analysis of the oxidative burst activity of granulocytes showed a statistically significant increase (*p* < 0.05) in the percentage of activated cells on day 4 following the first injection of pegbovigrastim when compared to the NC group. Similarly, on day 4 following the second injection of pegbovigrastim (day 11 of the study) statistically significantly higher (*p* < 0.05) values of this parameter for the E group than in the NC group were also observed, despite ongoing *M. bovis* infection. Apart from these two time points, no statistically significant differences (*p* < 0.05) between all examined groups in the percentage of activated granulocytes were observed until the end of the study on day 28 (Figure 7).

**Figure 8:**
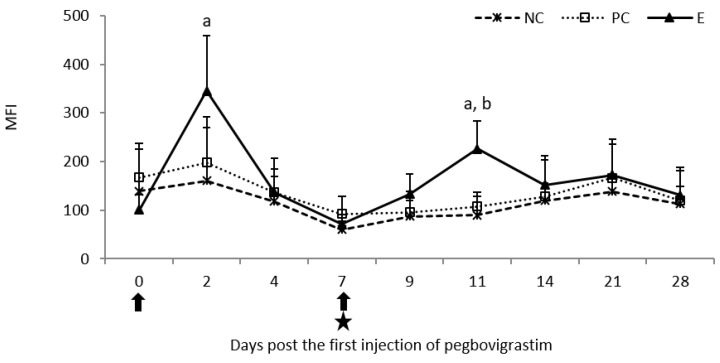
Oxidative burst activity of granulocytes expressed as mean fluorescence intensity (MFI) in the peripheral blood of calves after injection of pegbovigrastim and post-challenge with *M. bovis*. E—experimental group; PC—positive control group; NC—negative control group; arrow—pegbovigrastim injection; star—infecting dose of *M. bovis*; a—*p* < 0.05 between the E and NC groups; b—*p* < 0.05 between the E and PC groups.

The first injection of pegbovigrastim caused a statistically significant increase (*p* < 0.05) in the MFI as a result of the oxidative burst activity of granulocytes on day 2 when compared to the NC group. The increase continued after the second injection of pegbovigrastim, despite ongoing *M. bovis* infection, and it was statistically significant (*p* < 0.05) on day 11 of the study when compared to both control groups. At the remaining time points, the values of this parameter were comparable in all examined groups (Figure 8).

**Figure 9:**
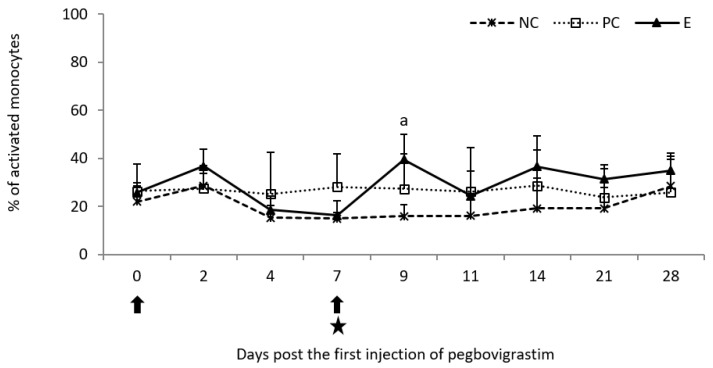
Oxidative burst activity of monocytes expressed as a mean percentage of cells in the peripheral blood of calves after injection of pegbovigrastim and post-challenge with *M. bovis*. E—experimental group; PC—positive control group; NC—negative control group; arrow—pegbovigrastim injection; star—infecting dose of *M. bovis*; a—*p* < 0.05 between the E and NC groups.

Analysis of oxidative burst activity of monocytes showed a slight increase in the percentage of activated cells on day 2 following the first injection of pegbovigrastim when compared to both controls. The increase continued after the second injection of pegbovigrastim, despite ongoing *M. bovis* infection, and it was statistically significant (*p* < 0.05) on day 9 when compared to the NC group. Higher, albeit not statistically significant (*p* < 0.05), values of this parameter were still observed in the E group on days 14, 21, and 28 when compared to both control groups (Figure 9).

**Figure 10:**
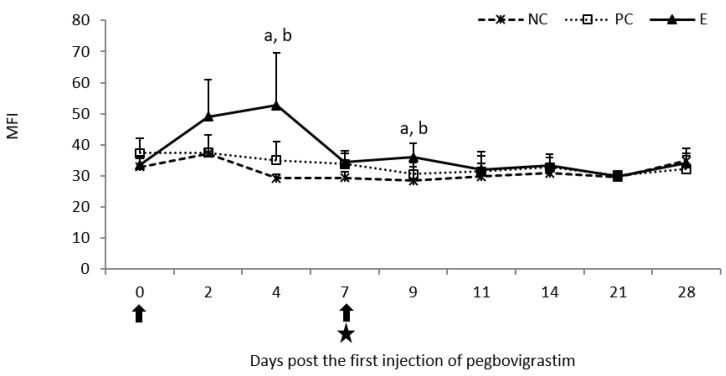
Oxidative burst activity of monocytes expressed as mean fluorescence intensity (MFI) in the peripheral blood of calves after injection of pegbovigrastim and post-challenge with *M. bovis*. E—experimental group; PC—positive control group; NC—negative control group; arrow—pegbovigrastim injection; star—infecting dose of *M. bovis*; a—*p* < 0.05 between the E and NC groups; b—*p* < 0.05 between the E and PC groups.

The first injection of pegbovigrastim caused a statistically significant increase (*p* < 0.05) in the MFI as a result of the oxidative burst activity of monocytes on day 4 when compared to both controls. On day 9 of the study (day 2 following the second pegbovigrastim injection), the increase was still statistically significant (*p* < 0.05) when compared to both controls despite ongoing *M. bovis* infection. At the remaining time points following the second injection of pegbovigrastim in the E group and the infection in the E and PC groups, the values of this parameter were comparable with those in the NC group (Figure 10).

## 3. Discussion

In this study, the effect of pegbovigrastim injection on the number of peripheral blood granulocytes and monocytes and their functions in the calves experimentally infected with *M. bovis* was investigated for the first time. Most available studies on pegbovigrastim have focused on reducing the occurrence of clinical mastitis and other diseases during the periparturient period in cows, while this study attempted to use pegbovigrastim in the prevention of respiratory diseases in calves, which are largely caused by *M. bovis*.

*M. bovis* is known to modulate the immune response of the host [4]. It was previously reported that *M. bovis* may affect bovine leukocytes and some functions of the cells, including proliferation, apoptosis, or oxygen-dependent microbicidal response [5,6,7]. Due to a lack of commercially available vaccines in Europe and increasing resistance of field *M. bovis* isolates to the commonly used antimicrobials, there is a need to look for new solutions to prevent infections with the pathogen [8,11].

Previous studies on adult cattle showed visible stimulation of the white blood cell response post-administration with pegbovigrastim, which was manifested by a significant increase in the number of circulating leukocytes including neutrophils and monocytes during the transition period [19,23]. Until now, only two studies investigating the effect of pegbovigrastim injection in calves were performed, one of which concerns the use of pegbovigrastim co-administered with enrofloxacin and flunixin meglumine in the treatment of *M. bovis* pneumonia [22,24]. The second of them evaluated the effect of pegbovigrastim injection on selected hematological and biochemical parameters in the calves under physiological conditions, as well as during LPS-induced endotoxemia [22]. In this study, a significant increase in the number of total leukocytes, segmented neutrophils, and monocytes on days 2 and 4 following administration of pegbovigrastim was investigated. The results of this study are consistent with our observations where a significant stimulation of the granulocyte and monocyte counts was observed up to day 7 following the first injection of pegbovigrastim. The magnitude and course of the leukocyte response were similar in both studies, despite the differences resulting from the age of the calves assigned to the experiment (newborn calves) and a pegbovigrastim dose of 25 µg/kg of BW which was lower than in our study. The duration of the response is difficult to compare fully because, on day 7 post pegbovigrastim administration in our study, the calves were infected with *M. bovis* and the dose of pegbovigrastim was repeated, while in the study of Kegles et al. [2019] the analyses were completed on day 4 post stimulation [22]. In another experiment by the same authors, the effect of a single injection of pegbovigrastim in 30–60 d old calves challenged with *Escherichia coli* LPS on the number of circulating leukocytes was investigated. The results showed a visible increase in the total leukocyte and segmented neutrophil counts until day 3 post administration of pegbovigrastim, despite the previous LPS injection [22]. In our study, the second injection of pegbovigrastim caused an enhancement of both the granulocyte and monocyte responses initiated by the first administration, even when the calves were infected with *M. bovis* which, in itself, decreased the number of cells. Neutrophils play a main role in pathogen phagocytosis which is an important mechanism of innate immunity, enabling an early response to the pathogen before adaptive immunity appears. Monocytes are also involved in phagocytosis and, as macrophages, they play an important role in the second line of the innate immune response. However, as antigen-presenting cells, they participate in initiating adaptive immunity [25]. In our study, the administration of pegbovigrastim caused a significant increase in the percentage of phagocytic granulocytes, persisting despite infection with *M. bovis* which, itself, caused some attenuation of this response. On the other hand, the MFI value for the phagocytic activity of granulocytes was visibly enhanced post second injection of pegbovigrastim and ongoing infection with *M. bovis*. At that time, some stimulation of this parameter was also observed in the infected-only group, but it was significantly lower than in the group additionally treated with pegbovigrastim. These results clearly indicate the beneficial effect of pegbovigrastim on the phagocytic activity of granulocytes in calves challenged with *M. bovis*, which is reflected not only in an increase in the number of cells capable of ingesting bacteria, but also in a significant increase in the individual cellular phagocytic activity of granulocytes. In our study, the phagocytic activity of monocytes was generally poorly expressed following injection of pegbovigrastim. An increase in the percentage of phagocytic monocytes was observed following the second injection of pegbovigrastim in the *M. bovis*-challenged calves when compared to the infected-only calves, in which some impairment of the overall phagocytic activity of monocytes was revealed.

Phagocytes are also able to kill bacteria by an oxygen-dependent mechanism, oxidative burst, resulting in reactive oxygen intermediate production [25]. In our study, the percentage of granulocytes able to produce reactive oxidants was increased especially post second injection of pegbovigrastim in the calves challenged with *M. bovis*. The stimulation of oxidative activity was more expressed in MFI values, which were significantly increased after the second administration of pegbovigrastim despite ongoing *M. bovis* infection. The infection somewhat inhibited this response, as it was slightly lower than that following the first injection of pegbovigrastim. However, the impairment of this response was not evident in the infected-only group.

The oxidative burst activity of monocytes expressed as a percentage of activated cells was visible post second pegbovigrastim administration despite ongoing infection with *M. bovis*. Compared to granulocytes, this response appeared earlier and was more evident. As for the MFI value which expresses the enzymatic activity of the cells, compared to the granulocytes, the stimulation of this activity in the monocytes lasted longer after the first injection of pegbovigrastim. On the other hand, post second administration of pegbovigrastim, in comparison to granulocytes, a significant stimulation of the MFI for monocytes appeared earlier; however, it was less expressed and lasted for a shorter time.

Pegbovigrastim administration in the calves significantly stimulated an increase in circulating granulocyte and monocyte number as well as phagocytic activity of the cells, especially granulocytes, which was also generally expressed over the course of *M. bovis* infection. In response to the administration, a general increase in the oxygen burst activity of the examined cells was observed. This effect was also shown despite ongoing infection with *M. bovis* in the calves, which altogether may indicate a beneficial effect of pegbovigrastim injection on the immunity of the affected animals. The use of pegbovigrastim in calves required a different approach, as it is intended for cows and used for a different purpose. Therefore, further studies are required to thoroughly evaluate the effect of pegbovigrastim on calves’ immunity, especially in preventing respiratory diseases, which are a major health problem in young stock.

## 4. Materials and Methods

### 4.1. Animals and Study Design

The study on animals was approved by the Local Ethics Committee on Animal Experimentation of the University of Life Sciences in Lublin, Poland (Decision no. 33/2018 admitted 12 Febuary 2018).

Eighteen clinically healthy female calves at the mean age of 7 weeks were purchased from local farmers and delivered to the Institute’s vivarium. The animals were free from *M. bovis* infection, which was confirmed by the examination of nasal and serum samples for antigen and specific antibody detection, respectively, using the Monoscreen AgELISA *Mycoplasma bovis* BIO K 341/2 and Monoscreen AbELISA *Mycoplasma bovis* BIO K 260/2, both manufactured by Bio-X Diagnostics S.A., Rochefort, Belgium.

After an adaptation period lasting 6 weeks, the calves were randomly assigned to three equal groups housed separately, experimental (E) and two controls: positive control (PC) and negative control (NC). The E group was subcutaneously injected with pegbovigrastim (Imrestor, Eli Lilly and Company Limited, Elanco Animal Health) at a single dose of 40 µg/kg of BW. The PC and NC groups were instead administered sterile phosphate-buffered saline pH 7.2 (PBS). On day 7 post pegbovigrastim administration, the E and PC groups were intratracheally infected with the *M. bovis* field strain KP795974 [4,24,26]. The inoculum concentration was 1.008 × 10^8^ CFU/mL. The method of preparation of the inoculum, which is a constant procedure used in the laboratory, has been described in detail previously by Dudek et al. [27]. The NC group instead received sterile PBS. Then, within 24 h after the infection, the E group was re-administered with pegbovigrastim at a single dose of 40 µg/kg of BW, while both control groups received sterile PBS instead. Blood samples were collected just before the first injection of pegbovigrastim/saline (day 0) and on days 2, 4, 7, 9, 11, 14, 21, and 28 after this injection.

### 4.2. Hematological and Flow Cytometry Analyses

Blood samples were collected from each animal by jugular vein puncture for assay of granulocyte (GRA) and monocyte (MON) counts and their functions as phagocytic and oxidative burst activities.

For hematological analysis, the samples were collected in a tube with K2-EDTA. The GRA and MON counts were calculated using a veterinary blood analyzer (Exigo, Boule Medical AB, Spånga, Sweden).

For flow cytometry analysis, the samples were collected in a tube with heparin. The quantitative determination of the phagocytic activity of peripheral blood granulocytes and monocytes was performed using a commercial kit (Phagotest™, Glycotope Biotechnology GmbH, Heidelberg, Germany) according to Wójcicka-Lorenowicz et al. [28] with modifications, and analyzed by flow cytometer (BD FACSCalibur, Becton Dickinson, Franklin Lakes, NJ, USA) using the CellQuest software (Becton Dickinson, Franklin Lakes, NJ, USA). The phagocytic activity of peripheral blood granulocytes and monocytes was expressed as the percentage of cells which ingest bacteria and mean fluorescence intensity (MFI) of the cells (number of bacteria per cell). The quantification of the oxidative burst activity of peripheral blood granulocytes and monocytes was performed using a commercial kit (Phagoburst™, Glycotope Biotechnology GmbH, Heidelberg, Germany) according to Wójcicka-Lorenowicz et al. [28] with modifications and analyzed by flow cytometer (BD FACSCalibur, Becton Dickinson, Franklin Lakes, NJ, USA) using the CellQuest soft-ware (Becton Dickinson, Franklin Lakes, NJ, USA). The oxidative burst activity of peripheral blood granulocytes and monocytes was expressed as the percentage of cells activated by *E. coli* which produce reactive oxidants and MFI for the determination of the enzymatic activity of the cells.

### 4.3. Statistical Analysis

The results are presented as arithmetic means or mean percentage ± standard deviation. The statistical analysis was performed using STATISTICA 10 software (StatSoft Inc., Tulsa, OK, USA). The differences between the mean values recorded in the experimental and control groups at the same time point were analyzed using one-way ANOVA with a statistically significant level of *p* < 0.05. To compare several groups against each other, Tukey’s post hoc test was performed.

## Data Availability

All the data supporting our findings are contained within the manuscript. The raw datasets analyzed during the current study are available from the corresponding author on reasonable request.
